# Retraction: Effect of supplemental parenteral nutrition on all-cause mortality in critically ill adults: a meta-analysis and subgroup analysis

**DOI:** 10.3389/fnut.2023.1345798

**Published:** 2023-12-01

**Authors:** Frontiers Editorial Office

The journal retracts the 22 August 2022 article cited above.

Following publication, concerns were raised regarding the misclassification of data used in the meta-analysis. An investigation, conducted in accordance with Frontiers' policies, confirmed that the data from the EPaNIC RCT, corresponding to approximately 60% of the study population of the meta-analysis, were misclassified. As the misclassification affects the results and conclusion of the article, the scientific validity of the article cannot be confirmed, and the article has been retracted.

This retraction was approved by the Chief Editor of Frontiers in Nutrition and the Chief Executive Editor of Frontiers. The authors do not agree to this retraction.

